# Picture quiz

**Published:** 2015

**Figure:**
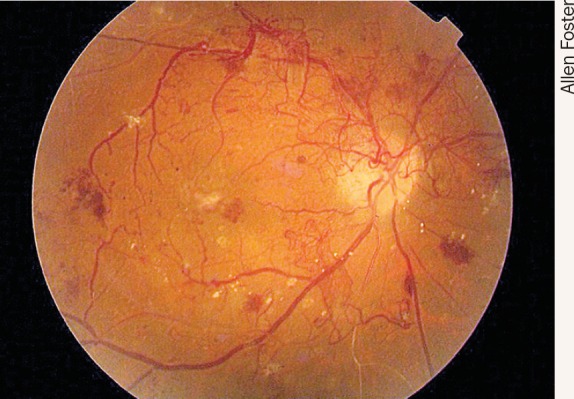


A 45-year-old man presents with blurring of vision in his good eye. The other eye has only light perception with a traction retinal detachment.

**Q1.** What is the most likely diagnosis?
  □ **a.** Papilloedema due to a brain tumour
  □ **b.** Proliferative diabetic retinopathy
  □ **c.** Hypertensive retinopathy
  □ **d.** Coat's disease
  □ **e.** Non-proliferative diabetic retinopathy
**Q2.** Which of the following clinical signs are present?
  □ **a.** Vitreous haemorrhage
  □ **b.** Neovascularisation of the retina
  □ **c.** Retinal haemorrhages
  □ **d.** Retinal hard exudates
  □ **e.** Retinal “cotton wool spots”

There may be more than one correct answer.

**Q3.** What treatments might be useful in managing this condition?
  □ **a.** Pattern laser treatment to the macular area
  □ **b.** Peripheral retinal photocoagulation
  □ **c.** Investigation of papilloedema
  □ **d.** Watch and review in 3 months
  □ **e.** Anti-vascular endothelial growth factor intra-vitreal injections

## ANSWERS

1. **Answer b.** This is severe proliferative diabetic retinopathy (PDR). The traction RD in the fellow eye is probably due to old PDR.
2. **Answer b, c, d and e.** In addition to haemorrhages and hard exudates, there are cotton-wool spots and widespread neovascularisation (new vessels) indicating retinal ischaemia.
3. **Answer b or possibly e if laser is not available.** The treatment of PDR is peripheral retinal photocoagulation (laser treatment). This needs to be done as soon as possible.

## REFLECTIVE LEARNING

Visit **www.cehjournal.org** to complete the online ‘Time to reflect’ section.

